# Loss of ubiquitin-conjugating enzyme E2 (Ubc9) in macrophages exacerbates multiple low-dose streptozotocin-induced diabetes by attenuating M2 macrophage polarization

**DOI:** 10.1038/s41419-019-2130-z

**Published:** 2019-11-26

**Authors:** Faxi Wang, Fei Sun, Jiahui Luo, Tiantian Yue, Longmin Chen, Haifeng Zhou, Jing Zhang, Chunliang Yang, Xi Luo, Qing Zhou, He Zhu, Jinxiu Li, Ping Yang, Fei Xiong, Qilin Yu, Huilan Zhang, Wanguang Zhang, Aimin Xu, Zhiguang Zhou, Qianjin Lu, Decio L. Eizirik, Shu Zhang, Cong-Yi Wang

**Affiliations:** 1 The Center for Biomedical Research, Key Laboratory of Organ Transplantation, Ministry of Education, NHC Key Laboratory of Organ Transplantation, Key Laboratory of Organ Transplantation, Chinese Academy of Medical Sciences,Tongji Hospital, Tongji Medical College, Huazhong University of Science & Technology, Wuhan, China; 2 Shenzhen Third People’s Hospital, 29 Bujibulan Road, Longgang District, 518000 Shenzhen, Guangdong China; 3 Department of Respiratory and Critical Care Medicine, Tongji Hospital, Tongji Medical College, Huazhong University of Science & Technology, Wuhan, China; 4 Department of Abdominal Surgery, Tongji Hospital, Tongji Medical College, Huazhong University of Science & Technology, Wuhan, China; 5 The State Key Laboratory of Pharmaceutical Biotechnology, The University of Hong Kong, 21 Sassoon Road, Laboratory Block, Pokfulam, Hong Kong China; 6 Department of Medicine, The University of Hong Kong, Hong Kong, China; 7 Department of Pharmacology and Pharmacy, The University of Hong Kong, Hong Kong, China; 8 Diabetes Center, The Second Xiangya Hospital, Institute of Metabolism and Endocrinology, Central South University, Changsha, China; 9 Department of Dermatology, Hunan Key Laboratory of Medical Epigenomics, Second Xiangya Hospital of Central South University, Changsha, China; 10 ULB Center for Diabetes Research, Université Libre de Bruxelles, Brussels, Belgium

**Keywords:** Phagocytes, Type 1 diabetes

## Abstract

Type 1 diabetes (T1D) is characterized by the selective autoimmune destruction of the islet β cells, and macrophages play a significant role in this process. Small ubiquitin-like modification (SUMOylation) is an important posttranslational modification involved in T1D pathogenesis, but its function in macrophages remains unexplored. We presently developed and used macrophage-specific ubiquitin-conjugating enzyme E2 (*Ubc9*) knockout (LyzM-Cre*-Ubc9*^fl/fl^, KO) mice to address the impact of SUMOylation on macrophage function in a T1D model. We observed that blocking Ubc9 in macrophages exacerbated multiple-low dose streptozotocin (MLD-STZ)-induced diabetes. Specifically, after STZ treatment, blood glucose levels were consistently elevated in the KO mice. The KO mice exhibited a higher diabetes incidence than WT controls (85% vs. 55%, *P* < 0.01) along with a higher insulitis severity. The loss of *Ubc9* impaired macrophage energy metabolism and attenuated macrophage M2 program, thereby enhancing T cell activation. Pancreas-resident macrophages, rather than migrant macrophages, played a predominant role in MLD-STZ-induced diabetes. Mechanistically, Ubc9-mediated SUMOylation of interferon regulator factor 4 (IRF4) enhanced its nuclear localization and stability, thereby transcribing IL-4 and arginase 1 (Arg1) to promote the macrophage M2 program. Ubc9-mediated SUMOylation modulates T1D risk at least in part by regulating macrophage function. Modulation of disturbed SUMOylation process in macrophages, either through cell adoptive transfer or targeted drug-delivery, could help to establish a tolerant pancreatic microenvironment and promote inflammation resolution in early insulitis stage, thus hindering T1D progression.

## Introduction

Immune-mediated islet inflammation (insulitis) is a key feature of type 1 diabetes (T1D), resulting in progressive islet β cell loss and eventually insulin dependence for life^1^. Therefore, a better understanding of the cellular components involved in insulitis and the underlying mechanisms leading to the attraction and activation of these cells is critical both to elucidate T1D pathogenesis and to develop novel immune-based intervention approaches^2^. Macrophages originated from the embryonic yolk sac and bone marrow reside in the pancreas from the time of birth^3^. During insulitis, they can also be recruited from the peripheral blood, and this cell population is called monocyte-derived macrophages^4^. Previous studies have shown that proper macrophage function is required for the maintenance of islet microenvironment and β cell function^5–7^, and that these closely regulated processes are altered during the early stages of insulitis. For instance, genetic predisposition renders macrophages in nonobese diabetic (NOD) mice with impaired phagocytosis^8–10^. As a result, β mass turnover in NOD neonates is associated with apoptotic β cell accumulation coupled with secondary necrosis and the release of damage-associated molecular patterns (DAMPs), such as HMGB1, contributing to the initiation of autoimmune responses against β cells^11–13^. Other than their phagocytic function, macrophages also function as professional antigen-presenting cells (APC) in the unique islet milieu^1,14^. Thus, macrophages establish residency in the pancreas before CD103^+^ dendritic cells (DCs) and may function as the earliest disease initiators.

Recent work has emphasized the role of alternatively activated macrophages, or M2 macrophages (mostly involved in tissue healing and repair, in contrast to the pro-inflammatory M1 macrophages), in the processes of insulitis and T1D^5,15^. Given that adoptive transfer of M2 macrophages attenuates insulitis progression, the reciprocal regulation between the islet β cell environment and alternative macrophage activation has therapeutic potential^16,17^. The activation of M2 macrophages is a tightly controlled cellular-programming process, depending on external factors, such as cytokines, intrinsic-signaling pathways, transcription factors and metabolic adaptation^18,19^. Despite past extensive studies, the key signals involved in M2 macrophage polarization in the context of diseases, such as T1D are yet to be fully elucidated.

SUMOylation mediated by the small ubiquitin-like modifiers (SUMO) is an evolutionarily conserved regulatory mechanism^20–22^. Dysregulation of ubiquitin-conjugating enzyme E2 (Ubc9)-mediated Nrf2 SUMOylation in β cells is linked to oxidative stress and β cells apoptosis^23^. We presently developed a macrophage-specific *Ubc9* knockout (KO) mice to address the impact of SUMOylation on macrophage function in a T1D model. We observed that ablation of SUMOylation impairs alternative macrophage activation and disturbs cellular energy metabolism. When challenged with multiple low-dose (MLD) of streptozotocin (STZ), a model of toxic and autoimmune diabetes^24^, the KO mice exhibited a severe disease phenotype that predominantly involved resident macrophages. The KO macrophages showed enhanced antigen uptake capacity, decreased glycolysis and oxidative phosphorylation (OXPHOS) along with attenuated M2 program and reduced capacity for regulatory T cell (Treg) induction, thereby promoting disease progression. Mechanistically, Ubc9-mediated SUMOylation of IRF4 was found to be essential for the M2 program in macrophages. Together, our data support that SUMOylation function in macrophages modulates T1D risk at least in part by regulating IRF4 stability and functionality along with cellular metabolic homeostasis.

## Materials and methods

### Animals

*Ubc9*^fl/fl^ mice (CD45.2) were generated as described previously^23^. These mice were backcrossed with LyzM-Cre mice (CD45.2, stock No.004781, Jackson’s Laboratory, Bar Harbor, ME, USA) to generate mice with a selective deletion of *Ubc9* in macrophages (LyzM-Cre-*Ubc9*^fl/fl^ mice), which were all heterozygous for LyzM-Cre. Age-matched and sex-matched 8–12-week-old littermates (*Ubc9*^fl/fl^) were used as controls, while no randomization method was applied. Wild-type (WT) CD45.1 congenic C57BL/6 and OT-II mice were purchased from the Jackson’s Laboratory (Bar Harbor, ME, USA). All mice were housed at the Tongji Medical College Animal Center with a 12/12-h light/dark cycle (Wuhan, China) in a specific pathogen-free (SPF) facility. All animal care and experimental procedures were approved by the Animal Care and Use Committee (ACUC) of Tongji Hospital and conducted in accordance with NIH guidelines. No specific statistical method was applied to determine the mouse number, and the blood glucose level was non-blindingly measured by the investigators.

### Antibodies and reagents

Recombinant murine IL-4(#214-14) and recombinant murine M-CSF (#315-02-250) were obtained from the PeproTech (Rocky Hill, USA), and STZ (#S0130) and 2-D08 (#SML1052) were purchased from the Sigma Aldridge (St. Louis, USA). Anti-Ubc9 (#4786), anti-IRF4 (#15106), anti-flag (#2368), anti-SUMO2/3 (#4971), anti-ubiquitin (#43124), anti-iNOS (#13120), anti-Arginase-1 (#93668), anti-PPAR-γ(#2443), anti-GFP (#2956), anti-HSL (#18381), and anti-p-HSL (Ser660) (#4126) antibodies were ordered from the Cell Signaling Technology (Danvers, MA, USA). The anti-CPT1a antibody (#15184-1) was obtained from Proteintech Group (Wuhan, China). PerCP-conjugated anti-mouse CD45 (#103130), PE-conjugated anti-mouse CD45.1 (#110708), APC-conjugated anti-mouse CD45.2 (#109814), FITC-conjugated anti-mouse F4/80 (#123108), PE-conjugated anti-mouse F4/80 (#123110), PerCP-conjugated anti-mouse F4/80 (#123126), PE-conjugated anti-mouse CD11b (#101207), APC-conjugated anti-mouse CD11c (#117310), FITC-conjugated anti-mouse CD206 (#141703), PE/Cy7-conjugated anti-mouse CD206 (#141720), APC-conjugated anti-mouse IL-4 (#504106), FITC-conjugated anti-mouse CD4 (#100406), PE-conjugated anti-mouse CD8 (#100708), PerCP-conjugated anti-mouse CD8 (#100732), PerCP-conjugated anti-mouse CD44 (#103036), APC-conjugated anti-mouse CD62L (#104412), APC-conjugated anti-mouse IL-17A (#506916), PE-conjugated anti-mouse IL-17A (#506904), APC-conjugated anti-mouse IFN-γ (#505810), PE/Cy7-conjugated anti-mouse IFN-γ (#505826), and Alexa Fluor®647-conjugated anti-mouse Foxp3 (#126408) antibodies were purchased from the BioLegend (San Diego, CA, USA).

### MLD-STZ-induced diabetes model

Mice (*n* = 20, each group) were given daily intraperitoneal injections of 50 mg/kg STZ dissolved in 0.1 M sodium citrate (pH 4.5) for 5 consecutive days. Blood glucose levels, body weight, and diabetes incidence were monitored every other day. Mice were defined as diabetic when their glucose levels were above 250 mg/dl for 3 consecutive days under non-fasting conditions^25^.

### RNA extraction and quantitative RT-PCR analysis

Total RNA was isolated from bone marrow-derived or peritoneal macrophages using the Trizol™ reagent (Takara, Japan). For mRNA analysis, an aliquot containing 1 μg of total RNA was reverse transcribed using a cDNA synthesis kit (Takara, Japan). Real-time PCR was performed using the SYBR Green PCR master mix (Applied Biosystems, South San Francisco, CA, USA) in the ABI Prism 7500 Sequence Detection System (Applied Biosystems, South San Francisco, USA). The following primers were used: arginase 1 (*Arg-1*) forward 5′-TTT TTC CAG CAG ACC AGC TT-3′, and reverse 5′-AGA GAT TAT CGG AGC GCC TT-3′; mannose receptor 1 (*Mrc-1*) forward 5′-CAG GTG TGG GCT CAG GTA GT-3′, and reverse 5′-TGG CAT GTC CTG GAA TGA T-3′; macrophage galactose-type lectin 1 (*Mgl1*) forward 5′-CAG GAT CCA GAC AGA TAC GGA-3′, and reverse 5′-GGA AGC CAA GAC TTC ACA CTG-3′; interleukin 6 (*IL-6*) forward 5′-ATG GAT GCT ACC AAA CTG GAT-3′, and reverse 5′-TGA AGG ACT CTG GCT TTG TCT-3′; interleukin 1β (*IL-1β*) forward 5′-GGA TGA GGA CAT GAG CAC CT-3′, and reverse 5′-GGA GCC TGT AGT GCA GTT GT-3′; tumor necrosis factor-α (*TNF-α*) forward 5′-ACT GAA CTT CGG GGT GAT CG-3′, and reverse 5′-GGC TAC AGG CTT GTC ACT CG-3′; and *β-actin* forward 5′-AGC CAT GTA CGT AGC CAT CC-3′, and reverse 5′-CTC CAG CTG TGG TGG TGA A-3′. The relative expression level of each gene was calculated with the 2^−ΔΔCt^ method as previously reported and normalized to the *β-actin* expression level^26^.

### Western blot analysis

Cell lysates were prepared using the radioimmunoprecipitation assay (RIPA) buffer (Servicebio, Wuhan, China) containing a protease inhibitor cocktail (Roche, IN, USA). Western blot analysis of target proteins was conducted as described using appropriate primary antibodies, followed by probing to the corresponding HRP-conjugated secondary antibody^27^. The reactive bands were visualized using ECL plus reagents (Servicebio, Wuhan, China), and the relative intensities of each band were analyzed using the ImageJ software.

### Cell culture

BMDMs were differentiated with M-CSF as previously reported^28^. The differentiated BMDMs were treated with 100 ng/ml LPS (Sigma, St. Louis, USA) or 10 ng/ml IL-4 or left untreated for the indicated periods of time. The cells were then harvested for quantitative RT-PCR, flow cytometry, and Western blot analyses. RAW264.7 cells (ATCC, #TIB-71) and BMDMs were cultured in Dulbecco’s modified Eagle’s medium (DMEM) (Gibco, Shanghai, China) supplemented with 10% fetal bovine serum (HyClone, Wuhan, China) and 1% antibiotics (penicillin/streptomycin) (Beyotime, Wuhan, China). Adenoviruses (Vector, IRF4-WT, and IRF4-K349R) were packaged by the Han Biotech Co., Ltd. (Shanghai, China). BMDMs were transduced with the empty control virus (Vector) or the adenovirus carrying the murine WT IRF4 (IRF4-WT) or IRF4-K349R mutant (IRF4-K349R) and treated with IL-4 (10 ng/ml) for further analysis. The RAW264.7 cells were authenticated by STR profiling and tested for mycoplasma contamination.

### Transwell migration assay

A total of 2 × 10^5^ BMDMs were seeded in inserts with 100 ng/ml LPS in the presence of 100 ng/ml C–C motif chemokine ligand 2 (CCL2) in the lower chamber. The next day, the inserts were washed and stained with crystal violet. The stained BMDMs were imaged and analyzed by microscopy (BX53, Olympus, Japan) at ×100 magnifications.

### Seahorse metabolic assay

Approximately 2–3 × 10^5^ BMDMs were plated in XF24 cell culture microplates (Seahorse Bioscience, Santa Clara, CA, USA) and treated with M2-polarizing stimuli for the indicated time points to analyze the extracellular acidification rate (ECAR) and the oxygen consumption rate (OCR). After stimulation, the medium was changed to XF assay medium according to the manufacturer’s instructions. The ECAR and OCR were assessed using an XF24 analyzer (Seahorse Bioscience, Santa Clara, CA, USA). These data were normalized to the total protein content.

### Confocal microscopy

BMDMs were cultured as described above, followed by transducing adenoviruses (IRF4-WT and IRF4-K349R) for 24 h. After washes, the cells were next induced with IL-4 for M2 polarization as described above. Nuclei were stained by DAPI, and IRF4 was labeled by an anti-IRF4 antibody, followed by the fluorescently labeled Donkey Anti-Mouse 594 IgG (H + L) secondary antibody (1:300, Jackson Immuno Research, PA, USA). The stained BMDMs were imaged and analyzed by confocal microscopy (FV1000, Olympus, Japan) at ×60 magnifications.

### In vitro SUMOylation assay

BMDMs were transduced with either IRF4-WT or IRF4-K349R, and then stimulated with IL-4 (20 ng/ml) overnight. After washes in ice-cold phosphate-buffered saline (PBS), the cells were lysed on ice for 30 min in an IP lysis buffer (50 mM Tris–HCl, pH 7.5, 150 mM NaCl, 1% NP-40, 5 mM EDTA, and 0.1% SDS) containing protease inhibitors (10 μg/ml aprotinin, 10 μg/ml leupeptin, and 1 mM PMSF), phosphatase inhibitors (5 mM sodium pyrophosphate and 1 mM Na_3_VO_4_) and 20 mM N-ethylmaleimide (Sigma, St. Louis, MO, USA). The cell lysates were precleaned with protein G agarose beads (GE Healthcare, New York, USA) for 1 h and then incubated with 5 µg anti-FLAG antibody overnight, and proteins were then immunoprecipitated for an additional 4 h at 4 °C with protein G beads. The samples were probed with the indicated antibodies (anti-SUMO1 and anti-SUMO2/3) for immunoblot analysis.

### Parabiosis model

Three pairs of 8-week-old CD45.1 (WT) and CD45.2 (LyzM-Cre^−^*Ubc9*^fl/fl^) male mice were ligated by matching longitudinal skin incisions on their flanks. Their elbows and knees were then joined with dissolvable sutures, and the incisions were closed with wound clips. Postoperative care included the administration of Buprenex for pain management, 5% dextrose, and 0.9% sodium chloride. Nutritional gel packs were provided in each cage, and antibiotics (Sulfatrim) were dissolved in the drinking water during the course of experiments. Two weeks later, the parabiotic mice were treated with STZ as described above. The mice were sacrificed to collect pancreatic macrophages around 14 days following STZ induction, by then they became diabetic.

### Antigen uptake assay

FITC-Dextran was incubated with BMDMs for one hour at 37 °C, and the reaction was terminated by washing with ice-cold PBS. Single cell suspensions were then prepared for flow cytometry analysis of the intensity of FITC-Dextran.

### Macrophage and T cell coculture

BMDMs were stimulated with LPS (100 ng/ml) for 24 h, and then washed with cold PBS to remove the supernatant. Naïve CD4^+^ T cells were isolated from WT mice using a Naïve CD4 T Cell Isolation Kit according to the manufacturer’s instructions (Miltenyi Biotech, San Francisco, CA, USA). Macrophages and T cells were mixed at a ratio of 1:5 with the addition of an anti-CD3 antibody (1 µg/ml) and cocultured for 3–5 days, followed by flow cytometry analysis of the Th1 and Th17 cell subsets. For analysis of antigen-specific T cell activation, BMDMs were pulsed with OVA (323–339) (Sigma, St. Louis, MO, SUA), followed by stimulation with IL-4 (10 ng/ml) or vehicle for 24 h. Naïve CD4^+^ T cells were isolated from OT-II mice and then cocultured with the above OVA-pulsed macrophages (1:5 macrophage to T cell ratio) in the presence of IL-2 (10 ng/ml) and TGF-β (5 ng/ml) for another 3–5 days to check the efficacy of Treg induction.

### Chromatin immunoprecipitation (ChIP) assay

ChIP assays were performed as previously reported^29^. Briefly, BMDMs were stimulated with IL-4 (20 ng/ml) for 4 h prior to crosslinking with 1% formaldehyde and subsequent sonication. The IRF4–DNA complexes were next immunoprecipitated using an anti-IRF4 antibody. The resulting DNA fragments then served as templates to amplify the regions containing the IRF4-binding motif within the IL-4 and Arg-1 promoters. The following primers were used: IL-4 forward 5′-CAC ACA CAC ACG AAT CTG AAT GAG-3′, and reverse 5′-GGC TTT CTC TGA CTT GTC TTC TGA-3′; and Arg1 forward 5′-AAA TGG GTT CTT CGG GTC AAA C-3′, and reverse 5′-TCT ACA CAC CAG CAG AGT GTC AGC-3′.

### Luciferase reporter assay

Mouse IL-4 and Arg1 promoter sequences (~2000 bp upstream of 5′-UTR) were PCR amplified and cloned into a pGL3 vector as reported^30^. The primers used for PCR are as follows: IL-4 promoter forward 5′-AAA TCA GCC ATT TCT CAG GCT TC-3′ and reverse 5′-TGA AAA CAG GAA CTG AAA TGC AC-3′; and Arg1 promoter forward 5′-TGG ACG CAA CAG TCA GCA ATC ACT G-3′ and reverse: 5′-CCC CCT TAT AAT CAG GTT GGA TCT G-3′. RAW264.7 cells were transfected with plasmids carrying the IL-4 and Arg1 promoters along with either WT-IRF4 or K349R-IRF4 using a Lipofectamine 3000 kit (Invitrogen, USA). After 24 h of transfection, the cells were stimulated with IL-4 (10 ng/ml) as above. Cell lysates were then prepared and subjected to analysis of reporter activity using a Dual-Luciferase Reporter Assay System Kit (Promega, Madison, WI, USA).

### Flow cytometry analysis of pancreatic macrophages

The common bile duct was exposed once the mice were sacrificed. The pancreas was inflated through the bile duct with a 30-G needle connected to a 5-ml syringe containing 3 ml of pre-cooled collagenase P solution (0.5 mg/ml). The pancreas was then removed and digested in a 37 °C water bath for 5–10 min. Digestion was stopped by adding 9 ml of precooled 10% Hank’s buffer, and the cell suspension was centrifuged to remove the collagenase P. The cell suspension was washed three times with 10% Hank’s buffer and resuspended in FACS buffer for further staining. The cells were incubated with fluorescently labeled antibodies on ice in the dark for 20 min and then washed with FACS buffer (2% BSA in PBS). For analysis of intracellular molecules, the cells were permeabilized and stained intracellularly as previously described^31^. The cells were analyzed using an LSR Fortessa flow cytometer (BD Biosciences, Franklin Lakes, NJ, USA) equipped with FACSDiva software. Data were further analyzed using the FlowJo software.

### Histological analysis

Pancreatic tissue was harvested and fixed in 4% paraformaldehyde and then embedded in paraffin. Sections were prepared and stained with H&E as previously reported^32^.

### Adoptive transfer

BMDMs were generated from BM cells of 8–12-week-old WT or KO mice, and polarized to M2 subtype as described earlier. A total two million WT and KO M2 macrophages were intra-peritoneally injected into 8-week-old WT mice in two dosages (Day-1 and Day-4). After the last transfer of BMDMs, all the mice were subjected to MLD-STZ induction of diabetes (Day 0). Mice were divided into three groups (Control, *n* = 9; WT-M2, *n* = 10; KO-M2, *n* = 10) and sacrificed 14 days following STZ induction.

### Statistical analysis

All in vitro experiments were conducted with at least three independent biological replications. The log-rank (Mantel–Cox) test was used to determine the differences of diabetes incidence between the groups. An unpaired Student’s *t*-test was used to determine the significant differences between two groups. Statistical analysis of the data was conducted using the GraphPad Prism 5 software (GraphPad Software Inc., San Diego, CA, USA), and the results were expressed as mean ± SEM. In all cases, a *P* value < 0.05 was considered statistically significant.

## Results

### Mice with Ubc9 deficiency in macrophages are highly susceptible to MLD-STZ-induced diabetes

**Fig. 1:**
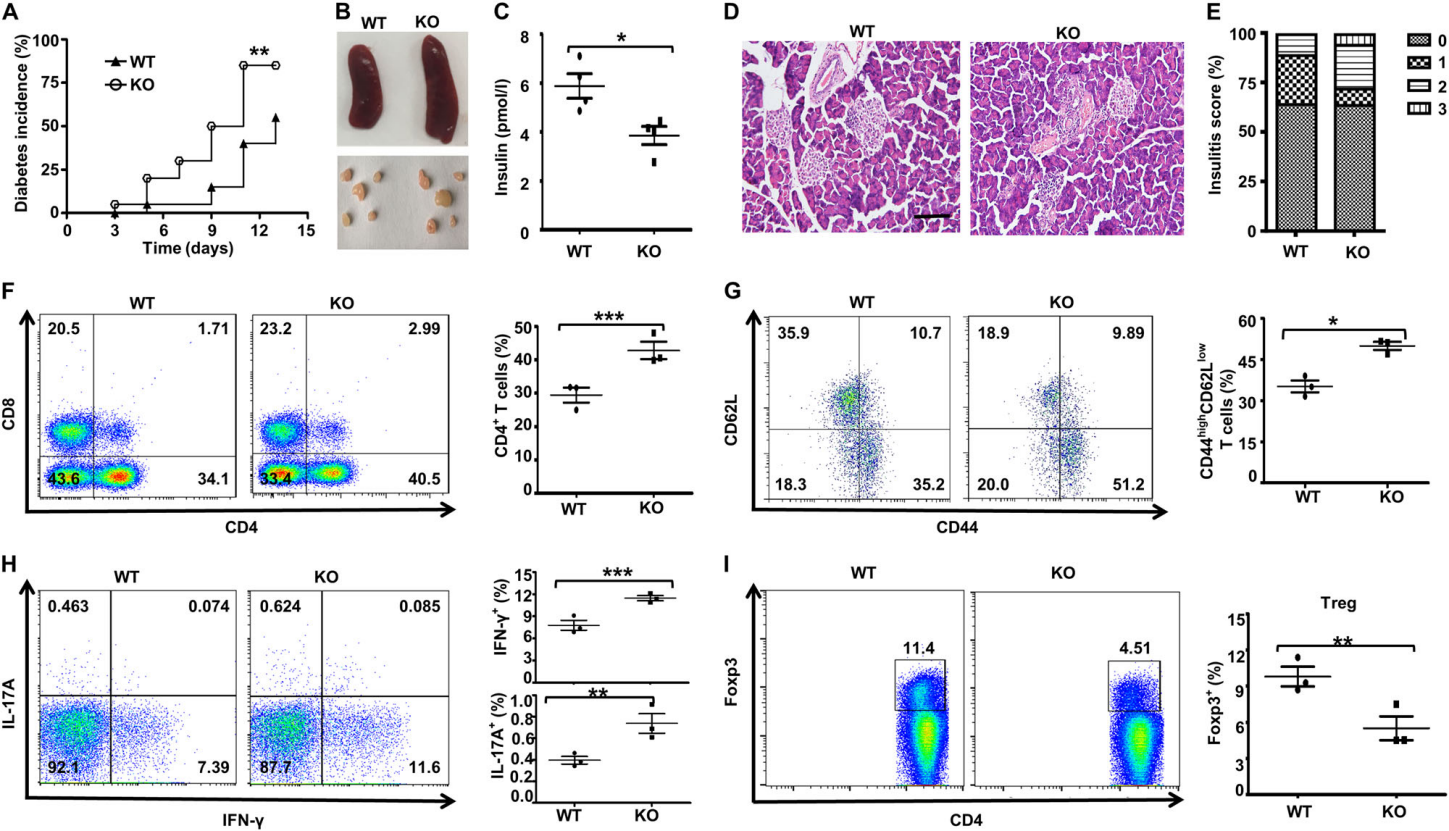
Macrophage-specific Ubc9 knockout mice have increased incidence of diabetes development following MLD-STZ treatment. Fourteen days after the initiation of STZ induction, the mice were sacrificed and used for the following experiments: Diabetes incidence curves **a** for the WT and KO mice (*n* = 20 for each group); Representative gross morphology of the spleen and pancreatic draining lymph nodes **b** of WT and KO mice; Plasma insulin concentrations **c** in diabetic WT and KO mice; Pancreatic tissue sections from diabetic WT and KO mice stained with H&E **d**, scale bar: 100 μm; and the insulitis score. **e** PLN cells from WT and KO mice (*n* = 4 for each group) were prepared and stained for flow cytometry. The percentages of CD4^+^, CD8^+^, CD4^+^CD44^high^ CD62L^low^, CD4^+^IL-17A^+^ (Th17), CD4^+^IFN-γ^+^ (Th1), and CD4^+^Foxp3^+^ (Treg) cells are shown in representative dot plots. Comparisons were carried out for the frequencies of CD4^+^ **f**, CD4^+^CD44^high^ CD62L^low^ **g**, Th1, Th17 **h**, and Treg cells **i**. Results are means ± SEM from three mice per group and are representative of three similar experiments conducted. *P < 0.05; **P < 0.01; ***P < 0.001; and ns, not significant.

To address the impact of *Ubc9* deficiency on diabetes, 8-week-old KO mice and littermates (*Ubc9*^fl/fl^, WT) were induced by MLD of STZ via intraperitoneal injections for 5 consecutive days, and non-fasting blood glucose levels and body weight changes were monitored every other day. Remarkably, the KO mice manifested significantly earlier diabetes onset (median incidence time 10 ± 0.83 days vs. 13 ± 1.8 days, *P* < 0.01) and higher diabetes incidence (85% vs. 55%, *P* < 0.01) than that observed in the control littermates (Fig. 1a). Although there were no differences in terms of body weight between the two groups of mice following STZ induction (data not shown), the KO mice showed splenomegaly and pancreatic lymphadenopathy (Fig. 1b), suggesting a more severe inflammatory response to the MLD-STZ. Consistently, there was a significant decrease in the plasma insulin level in the KO mice (Fig. 1c). Hematoxylin and eosin (H&E) staining of the pancreatic sections after day 14 of STZ induction indicated significantly higher inflammatory infiltration and islet destruction than that in control mice, which led to markedly higher insulitis scores in the KO mice (Fig. 1d, e).

We next compared the differences of T lymphocyte profiles in the pancreatic lymph nodes (PLNs) between KO and control mice at day 14 following STZ induction. The KO mice exhibited a higher proportion of CD4^+^ T cells than the control mice (Fig. 1f) and, to a lesser extent, CD8^+^ T cells. The proportion of CD4^+^CD44^high^CD62L^low^ activated T cells was significantly higher in the KO mice (Fig. 1g), and similarly, higher frequencies of both CD4^+^IFNγ^+^ (Th1) cells and CD4^+^IL-17A^+^ (Th17) cells were noted in the KO mice (Fig. 1h). In sharp contrast, the diabetic KO mice exhibited a 67% lower CD4^+^Foxp3^+^ Tregs than the WT mice (Fig. 1i).

### Ubc9 maintains the M1/M2 balance in the local pancreatic milieu

**Fig. 2:**
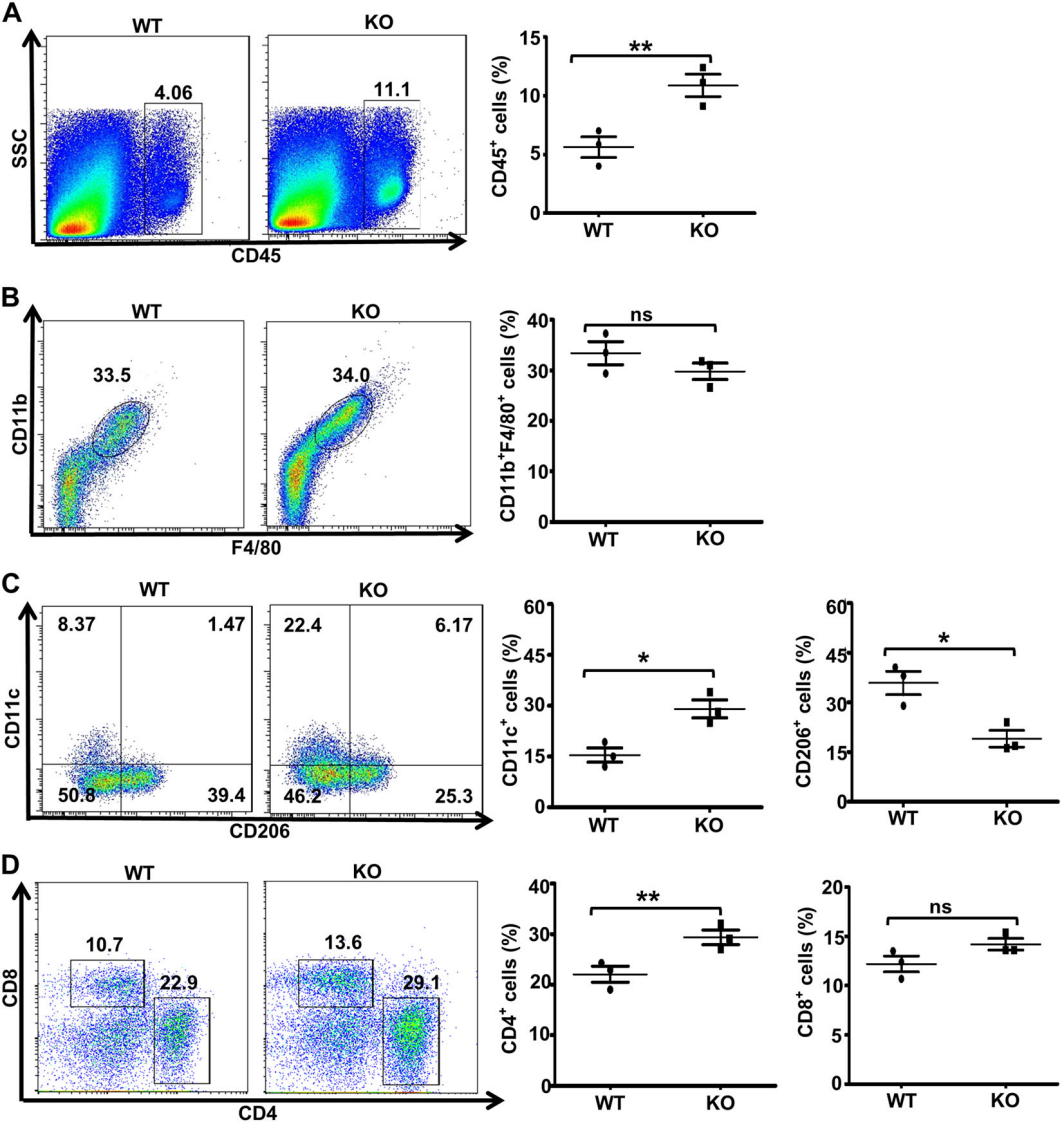
Macrophages in the local pancreatic milieu in MLD-STZ-treated mice. The frequencies of total macrophages, M1 macrophages and M2 macrophages were determined. Pancreatic cells from WT and KO mice (*n* = 4 for each group) were harvested on day 14 after STZ induction and stained with antibodies against CD45, CD11b, F4/80, CD11c, and CD206 for flow cytometry. The percentages of CD45^+^ **a**, CD11b^+^F4/80^+^ in CD45^+^ cells **b**, CD11c^+^ and CD206^+^ **c** in total macrophages and CD4^+^ or CD8^+^ T cells in CD45^+^ cells **d** are shown in representative dot plots. The frequencies of immune cells, total macrophages, CD11c^+^ or CD206^+^ macrophages and CD4^+^ or CD8^+^ T cells are shown in the bar chart. Results are means ± SEM from three mice per group and are representative of three similar experiments conducted. *P < 0.05; **P < 0.01; ***P < 0.001; and ns, not significant.

To evaluate whether local pancreatic macrophages were affected, pancreatic flow cytometry analysis was conducted. The KO mice displayed a 1.1-fold higher number of CD45^+^ cells in the pancreas than the control mice (Fig. 2a), which was consistent with the observation of higher severity of autoimmune responses. Although no difference was noted in terms of total (F4/80^+^CD11b^+^) macrophages (Fig. 2b), the percentage of CD206^+^ macrophages was significantly decreased in the KO mice, which was paralleled by a 1.3-fold higher CD11c^+^ macrophages (Fig. 2c). It was further noted that the frequency of CD4^+^ T cells, rather than CD8^+^ T cells, was increased in pancreas of KO mice, which may account for the local enrichment of CD45^+^ hematopoietic cells (Fig. 2d).

### Pancreas-resident macrophages play a dominant role in T1D development

**Fig. 3:**
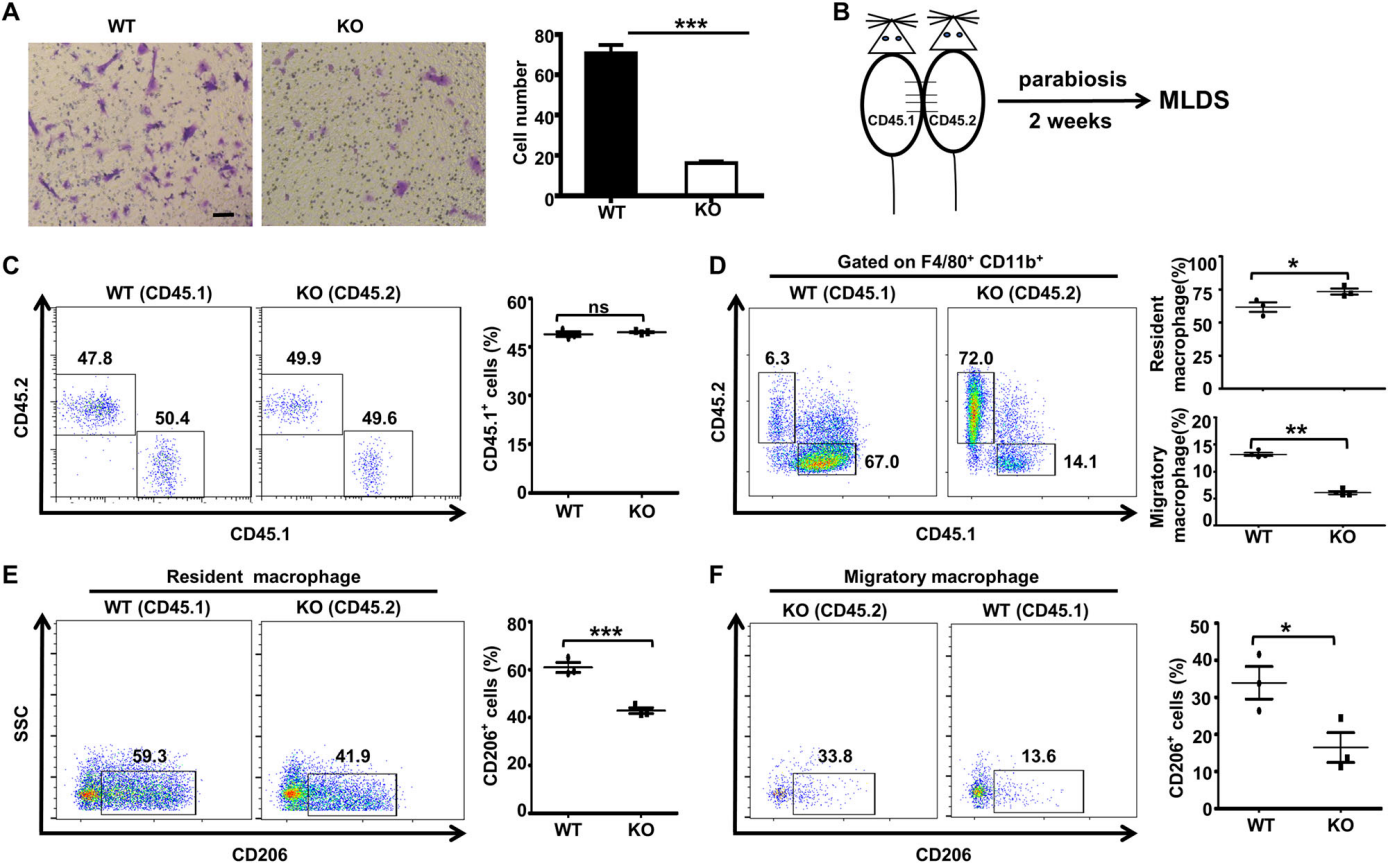
Pancreas-resident macrophages play a predominant role in the development of MLD-STZ-induced diabetes. WT and KO macrophage migration capacities were assessed in Transwell assays, and the cell numbers are shown in the bar graph **a**, scale bar: 50 μm. Wild-type (CD45.1) and KO (CD45.2) mice were used for parabiosis **b**. The proportions of CD45.1^+^ and CD45.2^+^ cells **c** in the peripheral blood were examined 2 weeks after surgical ligation. The percentages of CD45.1^+^ and CD45.2^+^ **d** cells in the CD11b^+^F4/80^+^ cell population from the ligated diabetic mouse pancreas 14 days after the initiation of STZ induction are shown in representative dot plots. The CD206^+^ macrophage subset of the resident macrophage population is shown in **e**. The frequency of CD206^+^ macrophages among migratory macrophages is shown in **f**. Results are means ± SEM from three mice per group and are representative of three similar experiments conducted. *P < 0.05; **P < 0.01; ***P < 0.001; and ns not significant.

During the inflammatory process, monocytes migrate from the blood into peripheral tissue and differentiate into macrophages (monocyte-derived macrophages). Hence, the pancreatic macrophages participating in insulitis could be derived from the circulation and/or originated from local resident macrophages activated by inflammation. To further dissect the effect of *Ubc9* deficiency on these macrophages from different origins, the transwell migration assay was carried out to assess macrophage migratory capacities. Surprisingly, the migratory capacity of KO cells was sharply reduced (Fig. 3a), indicating that the enrichment of macrophages in the pancreas may not be due to macrophages recruited from circulation in the KO mice.

To determine the origin of pancreatic macrophages in our MLD-STZ-induced disease model, parabiosis was performed (Fig. 3b). Two weeks after surgical ligation, we examined the proportion of immune cells in the peripheral blood between CD45.1(WT) and CD45.2(KO) mice. The percentages of CD45.1^+^ cells were nearly the same (Fig. 3c), demonstrating that our parabiosis model was successfully established. The mice were then treated with MLD-STZ, and blood glucose levels were monitored until the mice developed diabetes, followed by analysis of the pancreatic macrophages. In the WT mice, only 6.3% of the pancreatic macrophages (F4/80^+^CD11b^+^ gated) were derived from the KO mice, and similarly, only 14.1% of the pancreatic macrophages in the KO mice were originated from WT mice (Fig. 3d). Importantly, the predominant form in the pancreas was resident macrophages (nearly 70%) in both strains of mice (Fig. 3d), and the proportion of CD206^+^ macrophages in all resident macrophages was significantly lower in the KO cells than that observed in the WT cells (41.9% vs. 59.3%, *P* < 0.001, Fig. 3e). We then gated on the migrated pancreatic macrophages by analysis of CD45.1 and CD45.2 to examine their phenotype. Consistently, KO-derived CD45.2^+^ macrophages in the WT mice had a lower proportion of CD206^+^ macrophages as compared to that of WT-derived CD45.1^+^ macrophages in the KO mice (13.6% vs. 33.8%, *P* < 0.01; Fig. 3f), further confirming that *Ubc9* deficiency impairs the macrophage M1/M2 program in vivo.

### Ubc9 regulates macrophage cytokine profiles and antigen uptake

**Fig. 4:**
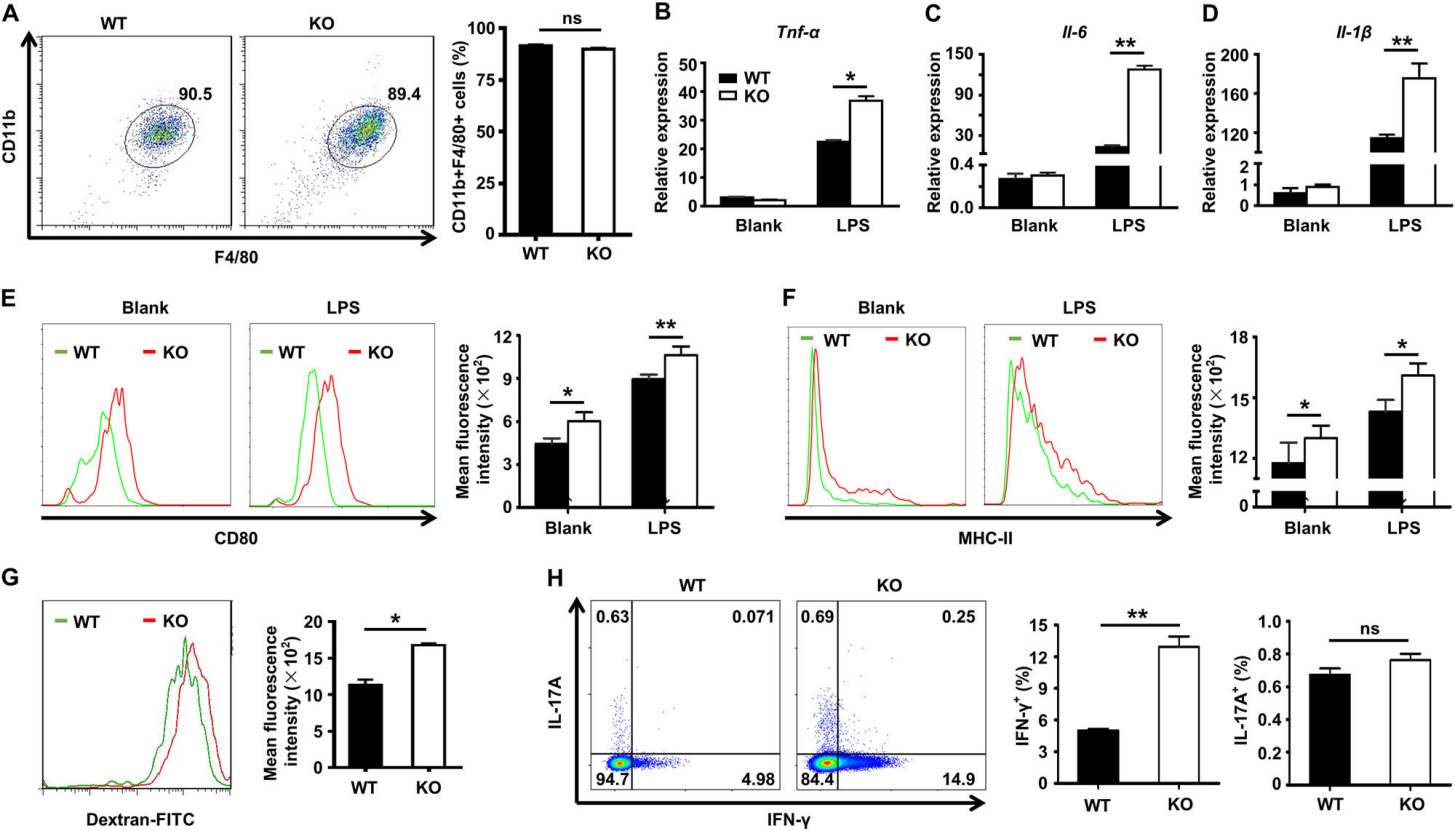
The impact of Ubc9 on macrophage proinflammatory cytokine production and antigen uptake capacity. Bone marrow cells were differentiated into macrophages and stained with antibodies against CD11b and F4/80, and the percentage of double-positive cells defined as the differentiation efficiency. **a** Real-time PCR analysis was conducted to assess *TNF-α* **b**, *IL-6* **c**, and *IL-1β* **d** mRNA expression in WT and KO macrophages with or without LPS treatment. Results were normalized to the expression of *β-actin*. Expression of CD80 **e** and MHC-II **f** is shown by histograms. Flow cytometry was used to quantify antigen uptake capacity based on the uptake of Dextran-FITC **e**. The results for T cell activation after coculture with macrophages are shown in **h**. Results are means ± SEM from three independent experiments conducted. *P < 0.05; **P < 0.01; ***P < 0.001; and ns not significant.

To further elucidate the impact of Ubc9 on macrophage functionality, we conducted in vitro experiments using bone marrow-derived macrophages (BMDMs). Interestingly, the loss of *Ubc9* did not result in a perceptible change in macrophage differentiation as evidenced by the similar frequency of CD11b^+^F4/80^+^ cells (Fig. 4a). However, once they were challenged by lipopolysaccharide (LPS), the KO BMDMs displayed much higher expression of proinflammatory cytokines, such as *TNF-α* (Fig. 4b), *IL-6* (Fig. 4c), and *IL-1β* (Fig. 4d). Consistently, the KO BMDMs displayed higher expression of iNOS, an M1 macrophage maker (Supplementary Fig. 2A), and similar results were obtained in peritoneal macrophages (Supplementary Fig. 2B). Moreover, the KO BMDMs expressed significantly higher levels of the co-stimulatory molecule CD80 (Fig. 4e) and MHC-II (Fig. 4f) than the control BMDMs.

The above results prompted us to check the capacity of antigen uptake in those KO cells. Indeed, KO BMDMs displayed markedly higher capacity for uptake of FITC-labeled dextran (Fig. 4g). Next, we checked their capability to induce CD4^+^ T cell activation. To this end, LPS-stimulated WT and KO BMDMs were co-cultured with naïve CD4^+^ T cells in the presence of an anti-CD3 antibody, followed by flow cytometry analysis of the production of Th1 (IFN-γ^+^) and Th17 (IL-17A^+^) cells. Unexpectedly, macrophages deficient in *Ubc9* did not affect their ability to induce Th17 cells, but significantly enhanced their capability to induce Th1 cells (Fig. 4h).

### Ubc9 deficiency impairs macrophage M2 program to affect Treg induction

**Fig. 5:**
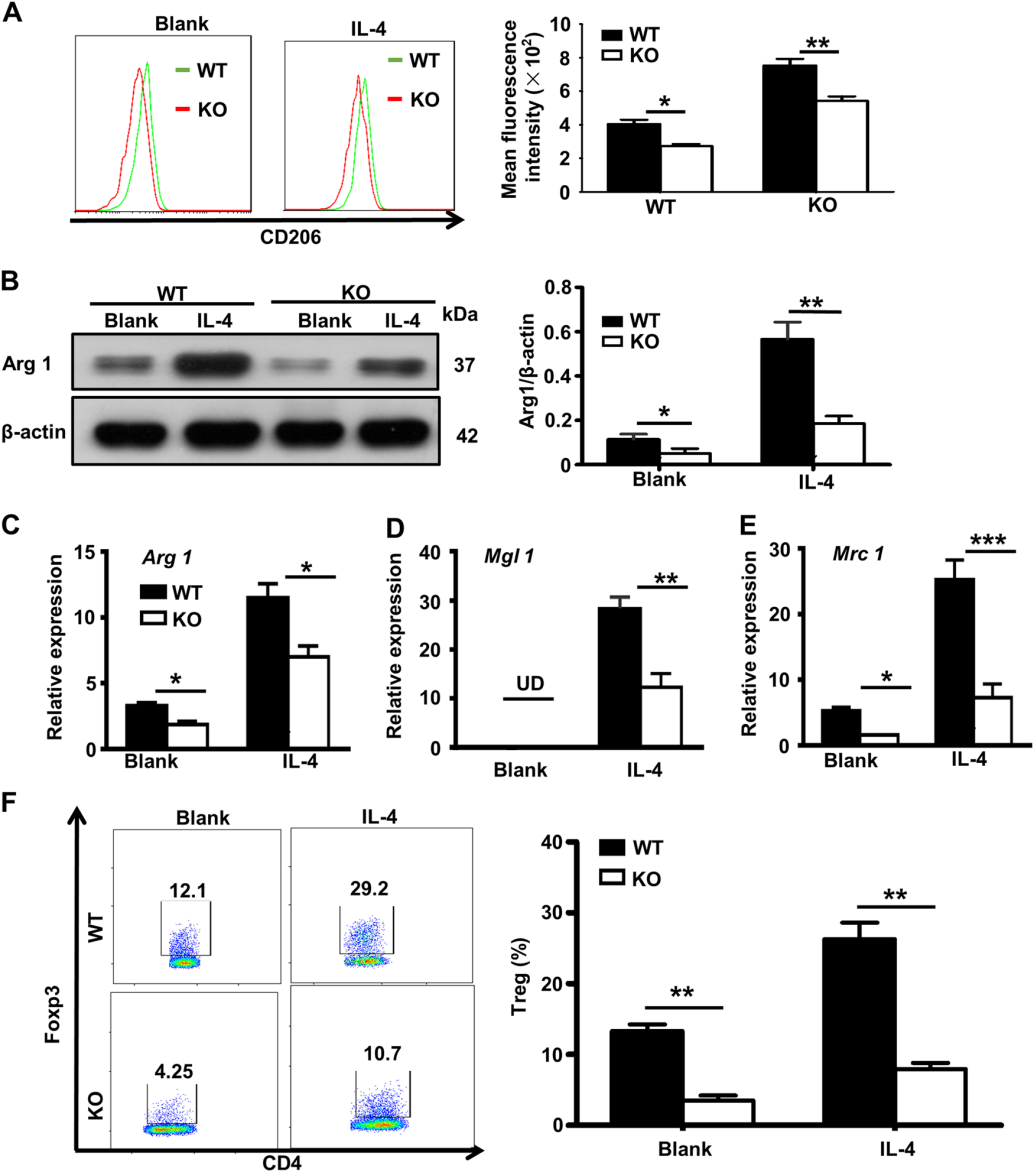
Ubc9 is required for IL-4-induced M2 macrophage polarization in vitro. BMDMs were treated with or without IL-4 and stained with antibodies against F4/80 and CD206, and the percentage of double-positive cells defined as the M2 macrophage proportion **a**. Immunoblotting was used to analyze Arg1 **b** expression in macrophages 24 h after IL-4 treatment. The intensities of the Arg1 protein signal relative to the intensity of the β-actin signal was shown in the bar graph. Real-time PCR analysis was conducted to assess *Arginase1* **c**, *Mgl1* **d**, and *Mrc1* **e** mRNA expression in WT and KO macrophages. Results were normalized to the expression of *β-actin*. Macrophages stimulated with IL-4 were cocultured with CD4^+^ T cells from OT-II mice in the presence of OVA (323–339) under Treg-differentiation conditions. The Treg-differentiation efficiencies are shown in representative plot dots and the bar graph **f**. Results are means ± SEM from three independent experiments conducted. *P < 0.05; **P < 0.01; ***P < 0.001; and ns, not significant.

To clarify the mechanisms by which mice with macrophages deficient in *Ubc9* have reduced Tregs (Fig. 1i), we first conducted studies to confirm that *Ubc9* deficiency impairs macrophage M2 program. In line with the results obtained in animals, KO BMDMs had significantly lower levels of CD206 expression following IL-4 induction (Fig. 5a). The attenuated macrophage M2 polarization was further confirmed by the decreased expression of molecules for the M2 program. Specifically, the loss of *Ubc9* led to a decrease of arginase 1 (Arg1) expression in response to IL-4 induction (Fig. 5b), and this result was further confirmed in peritoneal macrophages (Supplementary Fig. 1C) or by using 2-D08, an Ubc9 inhibitor (Supplementary Fig. 1D). Similarly, there were decreased mRNA levels for the M2 markers *Arg1* (Fig. 5c), macrophage galactose-type lectin 1 (*Mgl1*) (Fig. 5d) and mannose receptor 1 (*Mrc1*) (Fig. 5e) in the KO BMDMs following IL-4 induction. Next, we tested whether the impaired M2 program directly affects Treg induction. To this end, purified IL-4-induced M2 macrophages were pulsed with OVA (323–339), and then co-cultured with OT-II naïve CD4^+^ T cells in the presence of IL-2 and TGF-β for 3–5 days. Indeed, *Ubc9*-deficient M2 macrophages exhibited a 2.2-fold decrease in terms of the capability for induction of Tregs (Fig. 5f).

Next, adoptive transfer studies were conducted to explore the in vivo function of M2 macrophages. Eight-week-old mice were adoptively transferred with either WTM2 macrophages or KOM2 macrophages prior to MLD-STZ induction. Non-fasting blood glucose levels were monitored every other day following STZ treatment. As expected, adoptive transfer of WTM2 macrophages (WT-M2 group) rendered recipient mice with much lower diabetes incidence as compared to the control group (40% vs. 100%, *P* < 0.01; Supplementary Fig. 1A). Even though mice with adoptively transferred KO M2 macrophages (KO-M2 group) manifested lower diabetes incidence as compared to the control group (60% vs. 100%, *P* < 0.05), their disease incidence, however, was significantly higher than that of mice transferred with WT M2 macrophages (60% vs. 40%, *P* < 0.05; Supplementary Fig. 2A). H&E staining of the pancreatic sections confirmed that mice from the WT-M2 group were featured by the least inflammatory infiltration and islet destruction (Supplementary Fig. 2B). Of note, adoptive transfer of M2 macrophages led to a significant decrease of CD4^+^ T cells in the PLNs, but mice transferred with WT M2 macrophages displayed much lower number of CD4^+^ T cells (Supplementary Fig. 2C). Similarly, the percentage of activated CD4^+^ T cells (Supplementary Fig. 2D) and IFN-γ^+^ Th1 cells (Supplementary Fig. 2E) in the PLNs was much lower in mice from the WT-M2 group. In sharp contrast, the frequency of Tregs in the PLNs was the highest in mice transferred with WT M2 macrophages (Supplementary Fig. 1F). Together, our data suggest that macrophages deficient in *Ubc9* manifest impaired M2 program, thereby affecting Treg induction.

### Ubc9 regulates cellular metabolism essential for macrophage M2 program

**Fig. 6:**
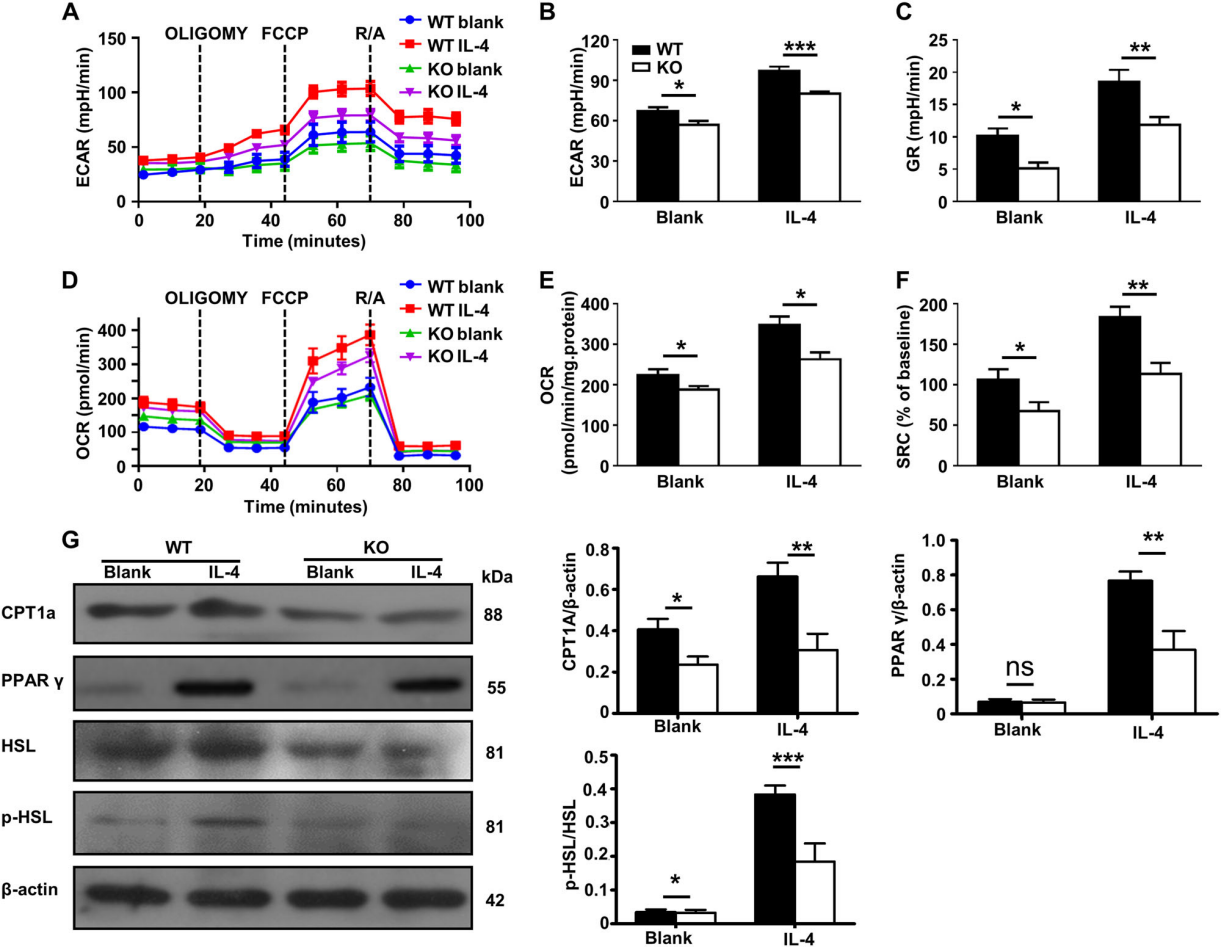
Ubc9 deficiency impairs glycolysis and mitochondrial respiration, which are essential for M2 polarization. ECARs at baseline and after sequential treatment with oligomycin (Oligo) and FCCP **a**, ECAR values **b**, and GR values **c** are shown in the bar graph. OCRs at baseline and after sequential treatment with oligomycin (Oligo) and FCCP **d**, OCR values **e**, and SRC values **f** are shown in the bar graphs. **g** Western blotting was used to analyze the protein expression of CPT1a, PPAR γ, and phosphorylated HSL. The intensities of the CPT1a, PPAR γ, and phosphorylated HSL protein signals relative to the intensity of the β-actin signal are shown in the bar graphs. Results are means ± SEM from three independent experiments conducted. *P < 0.05; **P < 0.01; ***P < 0.001; and ns not significant.

In contrast to M1 macrophages, M2 macrophages rely heavily on glycolysis and mitochondrial respiration, which includes OXPHOS and fatty acid oxidation (FAO)^33,34^. Given the role of Ubc9-mediated SUMOylation in the regulation of cellular metabolism^35–37^, we next checked whether *Ubc9* deficiency alters energy homeostasis, thereby impairing the M2 program. For this purpose, BMDMs were stimulated with IL-4 or control vehicle, and then subjected to the assessment of their ECAR (Fig. 6a) and OCR (Fig. 6d). There was a decreased ECAR (Fig. 6b) and glycolytic reserve (GR) (Fig. 6c) in the KO cells, indicating a reduced glycolysis. Interestingly, a decreased OCR (Fig. 6e) and spare respiratory capacity (SRC) (Fig. 6f) were also observed, suggesting that lipolysis was affected as well. To confirm this observation, we examined the expression of key enzymes responsible for lipolysis. The expression of carnitine palmitoyl transferase 1a (CPT1a), peroxisome proliferator-activated receptor γ (PPAR-γ), and phosphorylated hormone-sensitive lipase (HSL) were sharply decreased in the KO macrophages, confirming a repressed β-oxidation (Fig. 6g). Collectively, these data suggest that *Ubc9* deficiency impairs macrophage glycolysis and mitochondrial respiration, thereby attenuating the M2 program.

### Ubc9-mediated SUMOylation enhances IRF4 stability and functionality

**Fig. 7:**
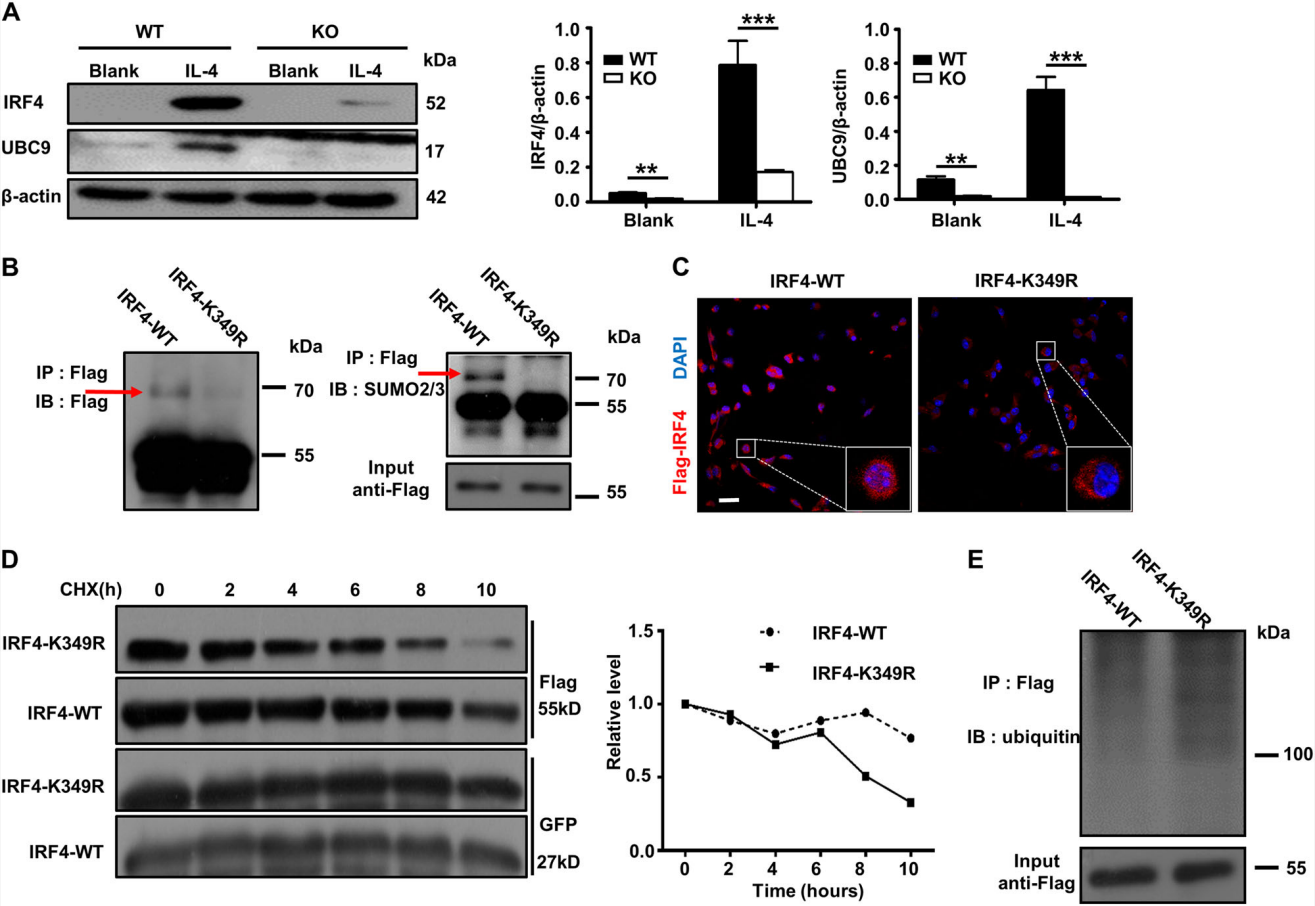
IRF4 is SUMOylated in macrophages and SUMOylation enhances IRF4 protein stability and nuclear translocation. **a** Western blotting was used to analyze IRF4 and Ubc9 expressions in macrophages 24 h after IL-4 treatment. The intensities of the IRF4 and Ubc9 protein signals relative to the intensity of the β-actin signal are shown in the bar graph. SUMOylated IRF4 was detected in macrophages transduced with Ad-IRF4-WT but not in Ad-IRF4-K349R transduced cells. Immunoprecipitates resulted from a Flag antibody were probed with an anti-Flag or an anti-SUMO2/3 antibody **b**, respectively. BMDMs transduced with Ad-IRF4-WT or Ad-IRF4-K349R were co-stained with an anti-flag antibody (red) and DAPI (blue) and imaged by confocal microscopy **c**, scale bar: 50 μm. CHX pulse-chase treatment was used to analyze the half-life of IRF4. Cells were treated with CHX for the indicated time points, and Western blot analysis was conducted to analyze the IRF4 protein levels **d**. **e** Results of an in vivo ubiquitination assay. The IRF4 immunoprecipitates were probed with an ubiquitin antibody for analysis of IRF4 ubiquitination. Results are means ± SEM from three independent experiments conducted. *P < 0.05; **P < 0.01; ***P < 0.001; and ns not significant.

To further dissect the mechanisms by which *Ubc9* deficiency impairs the macrophage M2 program, we next examined the impact of Ubc9-mediated SUMOylation on the functionality of IRF4, a key transcription factor that regulates M2 macrophage polarization through metabolic reprogramming^33,38^. We first checked the expression of IRF4 and Ubc9 in BMDMs following IL-4 induction. IRF4 was barely detectable and the expression of Ubc9 was quite low in WT BMDMs, while a 13-fold increased IRF4 expression coupled with a six-fold increased Ubc9 expression were noted following IL-4 induction (Fig. 7a). Interestingly, the potency for IL-4-induced IRF4 overexpression was decreased by four-fold in *Ubc9*-deficient BMDMs (Fig. 7a). IRF4 has been suggested to be a substrate for SUMOylation in T cells^39^, and we next examined whether Ubc9 mediates the SUMOylation of IRF4 in macrophages. IRF4 Flag-tagged WT IRF4 and IRF4-K349R (the lysine residue at position 349 was muted to arginine) were transfected into the BMDMs followed by IL-4 induction as above. Immunoprecipitation (IP) was then conducted using the anti-Flag antibody, and the resulting products were probed by the Flag or SUMO1 and SUMO2/3 antibodies. As expected, IRF4 was SUMOylated at lysine 349 in macrophages, not by SUMO1 (Supplementary Fig. 3A), but by SUMO2/3 (the SUMOylated form of IRF4 was indicated by an arrow, Fig. 7b). Of note, neither WT-IRF4 nor MU-IRF4 transduced Ubc9-deficient macrophages manifested detectable SUMOylated IRF4 (Supplementary Fig. 3B), and administration of 2-D08, an Ubc9 inhibitor, markedly decreased the SUMOylated IRF4 levels (Supplementary Fig. 3C).

To address the impact of Ubc9-mediated SUMOylation on IRF4 functionality, we transduced BMDMs with Ad-IRF4-WT or Ad-IRF4-K349R, and the transduced cells were subjected to IL-4 induction followed by immunostaining of IRF4 along with DAPI staining of the nuclei. Ablation of the IRF4 SUMOylation site (IRF4-K349R) significantly attenuated its nuclear localization following IL-4 induction (Fig. 7c). We further examined the impact of SUMOylation on IRF4 stability. BMDMs were transduced with the above adenoviruses before addition of cycloheximide (CHX) to inhibit IRF4 translation, respectively. The cells were then collected at the indicated time points for Western blot analysis of the IRF4 protein levels. Interestingly, the IRF4-K349R was more prone to degradation, with much lower protein levels following 8–10 h of CHX exposure (Fig. 7d). To further address this question, we examined IRF4 ubiquitination in transduced BMDMs, and the Ad-IRF4-K349R-transfected cells indeed displayed a higher level of the poly-ubiquitin-conjugated form of IRF4 than the Ad-IRF4-WT-transfected cells (Fig. 7e). Taken together, those data demonstrate that Ubc9 mediates SUMOylation of IRF4 in macrophages, by which it enhances IRF4 protein stability and its nuclear localization following IL-4 induction.

### SUMOylation facilitates IRF4 binding to transcribe IL-4 and Arg1 expressions

**Fig. 8:**
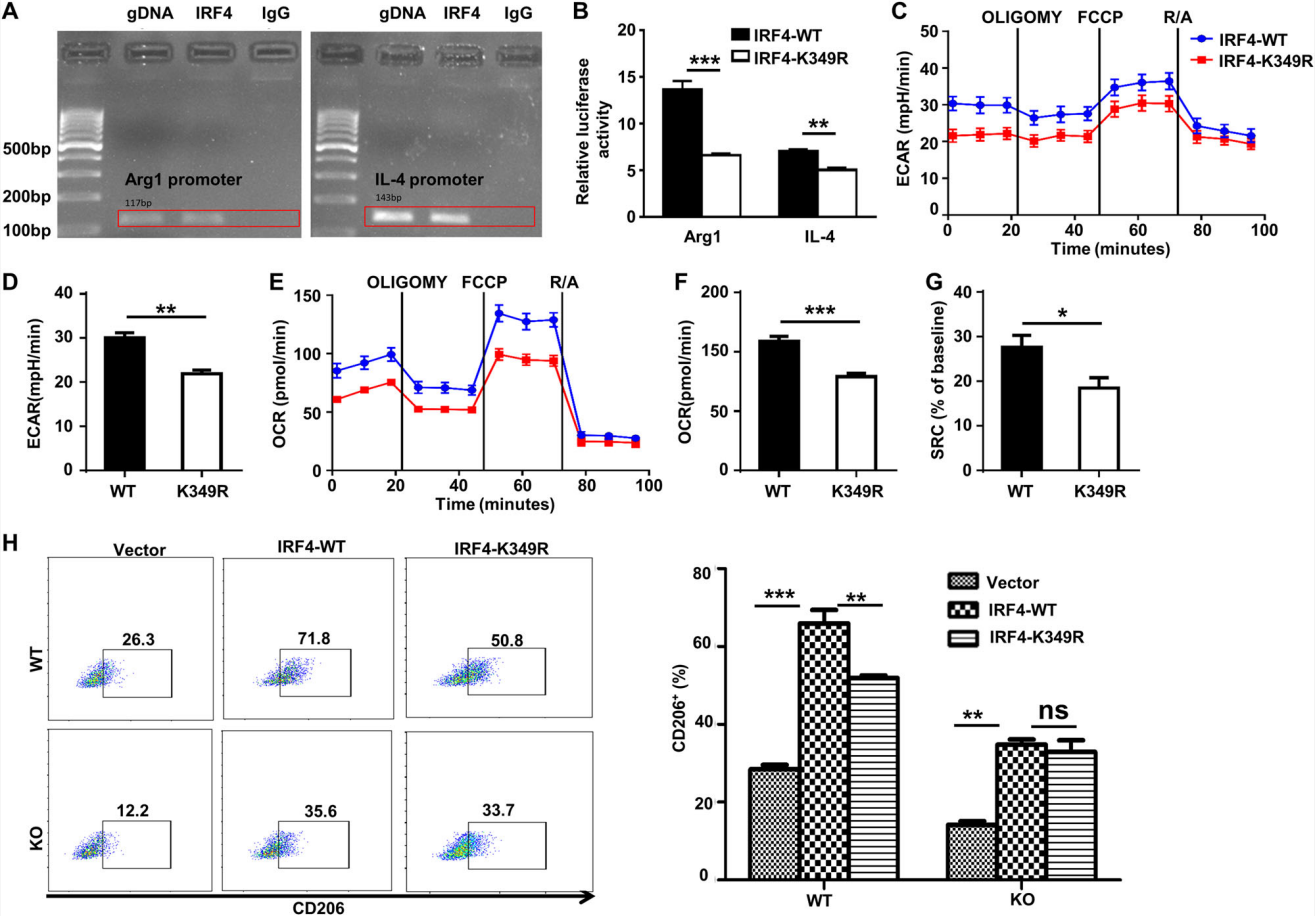
SUMOylation of IRF4 facilitates its binding to the IL-4 and Arg1 promoters and enhances macrophage M2 program. Chromatin immunoprecipitation (ChIP)-PCR results for IRF4 binding to the motif located in the *Il-4* promoter at position −303 bp and the motif in the *Arg1* promoter at position −361 bp are shown in **a**. Luciferase reporter activities in IL-4-stimulated RAW264.7 cells co-transfected with the *Il-4* or *Arg1* promoter reporter plasmid and the IRF4-WT or IRF4-K349R plasmid **b**. BMDMs were transduced with Ad-IRF4-WT or Ad-IRF4-K349R, and then stimulated with IL-4, followed by Seahorse and flow cytometry analyses. ECARs at baseline and after sequential treatment with oligomycin (Oligo) and FCCP **c** and ECAR values **d** are shown in the bar graphs. OCRs at baseline and after sequential treatment with oligomycin (Oligo) and FCCP **e**, OCR values **f**, and SRC values **g** are shown in the bar graphs. The frequencies of M2 macrophages transduced with Ad-IRF4-WT or Ad-IRF4-K349R either in WT or KO BMDMs are shown in representative plot dots and in the bar graph **h**. Results are means ± SEM from three independent experiments conducted. *P < 0.05; **P < 0.01; ***P < 0.001; and ns not significant.

The above results prompted us to further address the mechanisms by which SUMOylation facilitates IRF4 transcriptional activity to regulate M2 program in macrophages. We first conducted bioinformatic analysis of the IL-4 and Arg1 promoters with the MatInspector program, and one IRF4-binding site was found in each of the above two genes. To confirm experimentally these findings, ChIP assays were carried out in BMDMs, and the resulting products were employed for ChIP-PCR analysis of the regions flanking the above indicated putative IRF4-binding sites. IRF4 showed high binding activity to the motifs located at position −303 bp in the IL-4 promoter and at position −361 bp in the Arg1 promoter (transcription start site as +1, Fig. 8a). Promoter reporter assays were next conducted to address the role of SUMOylation in facilitating IRF4 binding to transcribeIL-4 and Arg1 expressions. As expected, BMDMs transduced with IRF4-K349R displayed significantly lower reporter activity for both IL-4 and Arg1 promoters (Fig. 8b). The next key question was whether SUMOylation facilitates IRF4 transcriptional activity by regulating cellular metabolism. BMDMs transduced with IRF4-K349R had significantly lower ECAR (Fig. 8c, d), OCR (Fig. 8e, f), and SRC values (Fig. 8g), which were consistent with the results obtained with the *Ubc9*-deficient macrophages (Fig. 6a, d). To address whether Ubc9 indeed regulates the M2 program via SUMOylation of IRF4, BMDMs were transduced with Ad-IRF4-WT or Ad-IRF4-K349R and then subjected to IL-4 induction for 24 h, followed by flow cytometry analysis by gating on GFP^+^ cells. Of note, the proportion of M2 (CD206^+^) macrophages in the Ad-IRF4-K349R-transduced BMDMs was decreased by 30% as compared to that of Ad-IRF4-WT-transduced BMDMs (Fig. 8h). Importantly, no perceptible difference was noted in terms of the production of M2 macrophages between Ad-IRF4-WT and Ad-IRF4-K349R-transduced KO BMDMs (Fig. 8h), confirming that Ubc9-mediated SUMOylation regulates IRF4 activity. Collectively, our data support the hypothesis that Ubc9 regulates the M2 program in macrophages at least in part through SUMOylation of IRF4, which enhances IRF4 stability and transcriptional activity by modulating metabolic homeostasis.

## Discussion

Immune cells exert systematic regulatory function, but in recent years their roles in maintaining the homeostasis of microenvironments in the organs and tissues have now increasingly been appreciated. Indeed, macrophages are critically important in local tissue homeostasis^40–42^, while its activation state is often associated with disease^19^. Specifically, the pancreas possesses a unique microenvironment, in which M1 macrophages localize within the islets, while M2 macrophages are mostly present in the interacinar stroma^3^. Once β-cell damage occurs in the early stages of T1D, the M1 macrophages within the islets can act as professional APC to initiate autoimmunity in genetically predisposed individuals and recruit CD103^+^ DCs into the islets. These DCs then migrate to the draining lymph nodes and activate lymphocytes, further amplifying the autoimmune reaction. On the other hand, the surrounding M2 macrophages promote islet formation during development and provide a microenvironment favorable for tolerance induction, inflammation resolution and tissue homeostasis under both physiological and pathological conditions^11,43^. Therefore, maintaining the homeostasis of M1 and M2 macrophages is crucial to the outcome of islet inflammation.

Previous studies have consistently demonstrated that altered SUMOylation function contributes to T1D pathogenesis^21,44–46^. Indeed, SUMOylation regulates cellular stress responses and plays an essential role for β-cell viability and functionality^23,47,48^. Particularly, SUMOylation has been suggested to affect the function of macrophages in the context of LPS-stimulated cytokine production and the antiviral response^32,49^. However, the effects of Ubc9-mediated SUMOylation on the macrophage M2 program and its implication for T1D pathogenesis remain to be elucidated.

In the present study, we observed an increased incidence in MLD-STZ-induced diabetes in mice with depletion of Ubc9 in their macrophages. This was accompanied by the disturbed T-cell subsets in the PLN. A significant decrease in the proportion of M2 macrophages was also noted among the total pancreatic macrophages in these KO mice following MLD-STZ challenge, although the total number of pancreatic macrophages remained unaltered. Given the fact that *Ubc9*-deficient macrophages manifest attenuated migratory capability, parabiosis was then carried out to determine the origins of those pancreatic macrophages during the course of diabetes development following MLD-STZ induction. Interestingly, we found that the enrichment of pancreatic macrophages was predominantly through proliferation of resident macrophages instead of recruitment of peripheral monocyte-derived macrophages. Importantly, the *Ubc9* deficiency significantly impaired the macrophage M2 program as evidenced by the remarkable reduction of M2 macrophages in the pancreas following MLD-STZ treatment.

The overall macrophage functional balance is achieved through the fine counter-regulation of the M1 and M2 subsets^19^. In line with this, *Ubc9*-deficient macrophages exhibited less potency to polarize to M2 macrophages upon IL-4 stimulation, and therefore, possessed a higher capability to uptake antigens and to stimulate T cell activation. Remarkably, M2 macrophages deficient in *Ubc9* also exhibited lower capacity for Treg induction. It has been suggested that energy metabolism including glycolysis and mitochondrial respiration, differentially affects macrophage subsets^18,34^. Among these subsets, the M2 subset is particularly sensitive to metabolic alterations in glucose and lipid metabolisms^50,51^. Intriguingly, we observed decreased glycolysis, OXPHOS and FAO levels in the *Ubc9*KO macrophages, which likely contributes to the observed impaired M2 program in these macrophages.

To explain how *Ubc9* deficiency alters macrophage energy metabolism, we focused on IRF4, a transcription factor that is highly expressed in M2 macrophages and plays an essential role to maintain the homeostasis of key metabolic processes, such as glycolysis and OXPHOS. We first confirmed that IRF4 is SUMOylated in macrophages at lysine 349 (K349), and further demonstrated that Ubc9-mediated SUMOylation of IRF4 both promotes its nuclear localization and enhances its stability. Indeed, the IRF4-K349R mutant had significantly lower binding activity to the *Il-4* and *Arg1* promoters, coupled with reduced capacity for induction of M2 macrophages. Next, we conducted studies by using IRF4-WT and IRF4-K349R adenoviruses, and confirmed that the IRF4-K349R mutant indeed alters macrophage metabolic characteristics. Taken together, our data support the hypothesis that Ubc9-mediated SUMOylation of IRF4 regulates macrophage energy metabolism, thereby playing an essential role in M2 macrophage polarization.

The exciting findings described above open several new areas of investigation. First, we observed decreased macrophage migration in the *Ubc9*-deficient macrophages. Unlike DCs, macrophages do not migrate into the draining lymph nodes, rather they interact with other cell types including T cells in the local sites^52^. We presently focused on the resident macrophages and their related polarization program, but it will be of future interest to clarify the mechanisms leading to the impaired migratory capacity. Given the facts that Rac1 is a SUMOylation target relevant to cell mobility^53^ and that ablation of its SUMOylation is associated with reduced cell movement in other cell types, such as tumor cells and synoviocytes^54^, the impaired migratory capability in the KO macrophages could be caused by the lack of Rac1 SUMOylation. Second, in the present study we focused on the effects of Ubc9-mediated SUMOylation of IRF4, a key transcription factor for the M2 program in macrophages. It is possible that additional SUMOylation targets also contribute to macrophage functional regulation. Therefore, future experiments using mass spectrometry and other advanced tools will be necessary to uncover the full complexity of SUMOylation in the regulation of macrophage polarization and functionality.

In summary, we have presently demonstrated that mice with macrophages deficient in *Ubc9* are prone to MLD-STZ-induced diabetes. Mechanistic studies revealed that Ubc9 plays an essential role for the macrophage M2 program, which at least in part involves SUMOylation of the transcription factor IRF4. Ubc9 mediates SUMOylation of IRF4 at lysine 349, through which it promotes IRF4 nuclear localization and stability, thereby regulating the homeostasis of energy metabolism favoring the M2 program in macrophages. As a whole, the present data brings novel insights into the mechanisms by which SUMOylation regulates M2 polarization in macrophages, and highlights the potential clinical significance of this evolutionarily conserved post-translational modification system in the pathogenesis of autoimmune diseases such asT1D.

## Supplementary information

Figure S1

Figure S2

Figure S3

Supplementary Figure caption

Reproducibility Checklist
