# Supplementary figures and images for: Loss of ubiquitin-conjugating enzyme E2 (Ubc9) in macrophages exacerbates multiple low-dose streptozotocin-induced diabetes by attenuating M2 macrophage polarization
Source: Cell Death Dis. 2019 Nov 26;10(12):892. doi: 10.1038/s41419-019-2130-z (PMC6877645; doi:10.1038/s41419-019-2130-z)

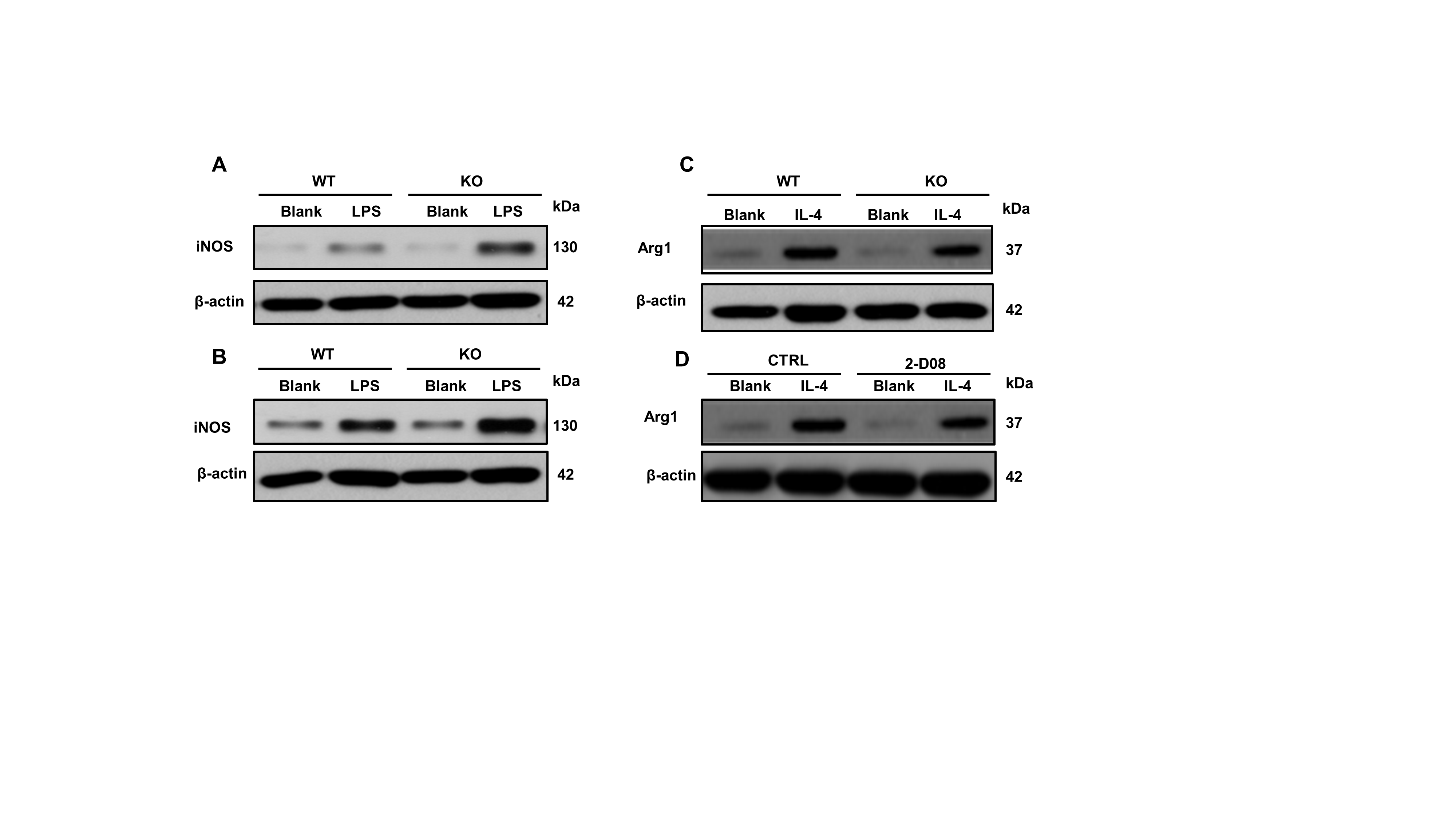

Supplement: Supplementary file 1 — Figure S1 [file 41419_2019_2130_MOESM1_ESM.tif]

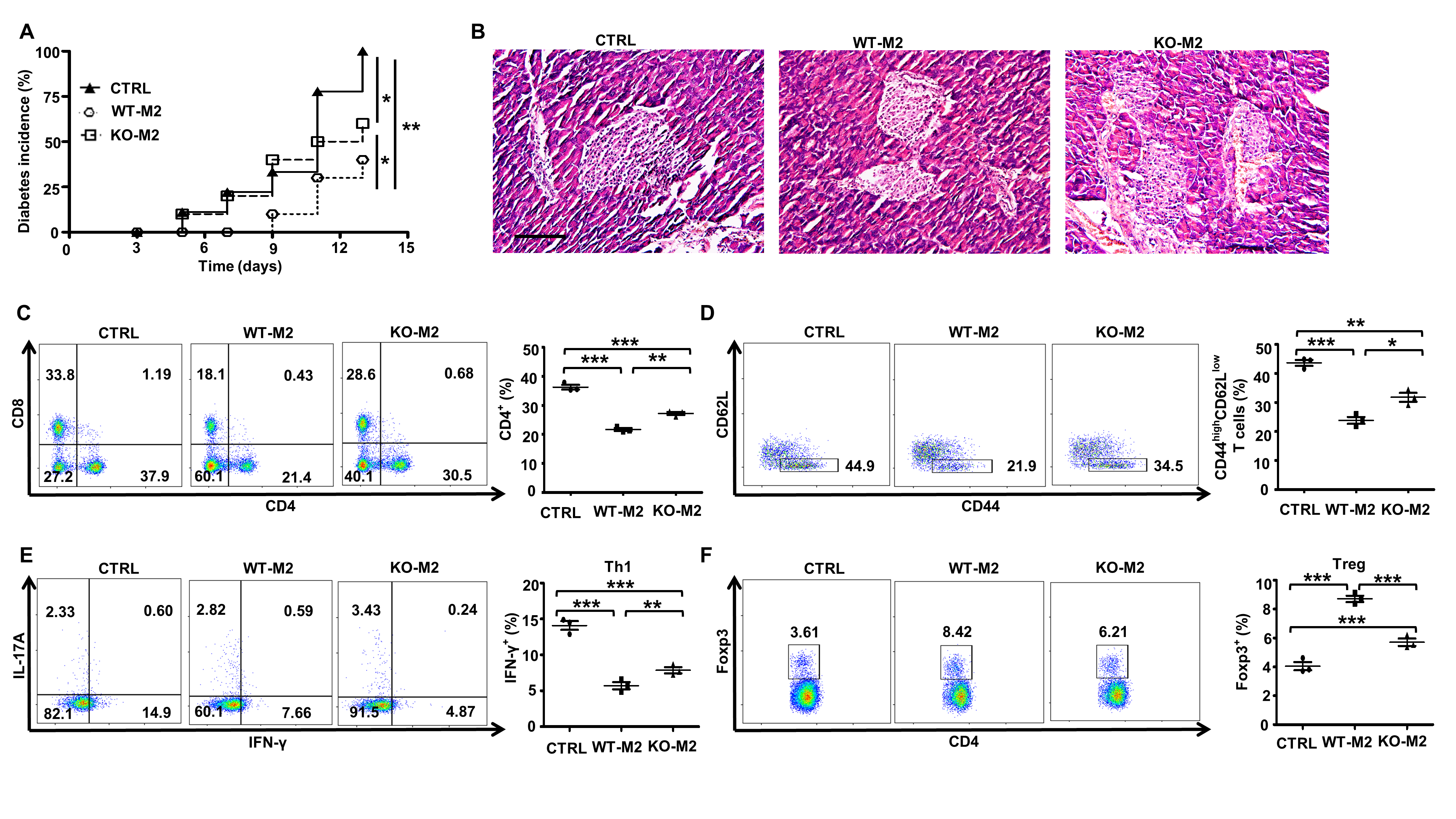

Supplement: Supplementary file 2 — Figure S2 [file 41419_2019_2130_MOESM2_ESM.tif]

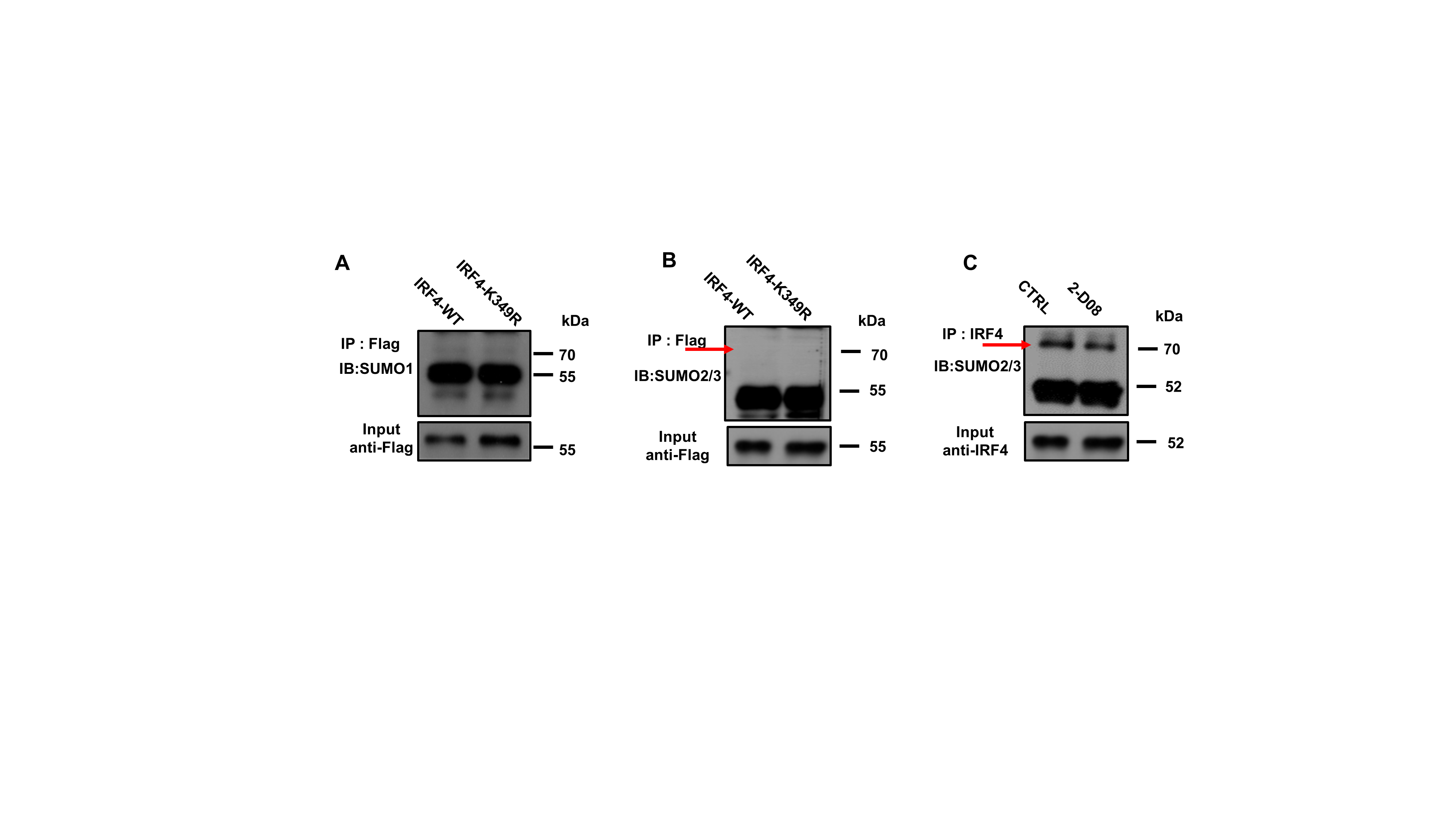

Supplement: Supplementary file 3 — Figure S3 [file 41419_2019_2130_MOESM3_ESM.tif]
