# Supplementary material for: Loss of ubiquitin-conjugating enzyme E2 (Ubc9) in macrophages exacerbates multiple low-dose streptozotocin-induced diabetes by attenuating M2 macrophage polarization
Source: Cell Death Dis. 2019 Nov 26;10(12):892. doi: 10.1038/s41419-019-2130-z (PMC6877645; doi:10.1038/s41419-019-2130-z)
Supplement: Supplementary file 5 — Reproducibility Checklist [file 41419_2019_2130_MOESM5_ESM.pdf]

DECLARATION OF CONTRIBUTIONS TO ARTICLE

ADMC

Manuscript Number:	Journal Name:	
19-2748	*Cell Death & Disease*	(the 'Journal')

Proposed Title of the Contribution:

Loss of ubiquitin conjugating enzyme E2 (Ubc9) in Macrophages Exacerbates Multiple Low-Dose Streptozotocin-Induced Diabetes by Attenuating M2 Macrophage Polarization (the 'Contribution)

Author(s):

Faxi Wang, Fei Sun, Jiahui Luo, Tiantian Yue, Longmin Chen, Haifeng Zhou, Jing Zhang, Chunliang Yang, Xi Luo, Qing Zhou, He Zhu, Jinxiu Li, Ping Yang, Fei Xiong, Qilin Yu, Huilan Zhang, Wanguang Zhang, Aimin Xu, Zhiguang Zhou, Qianjin Lu, Decio L. Eizirik, Shu Zhang, Cong-Yi Wang (the 'Authors')

For all *CDDis* articles, each person named as an author in the published version must be able to show he or she has contributed substantially to the article.

Authorship credit should be based on 1) substantial contributions to conception and design, acquisition of data, or analysis and interpretation of data; 2) drafting the article or revising it critically for important intellectual content; and 3) final approval of the version to be published. Authors should meet conditions 1, 2 and 3.

Any person who cannot be shown to have made a substantial contribution to the article cannot be listed as an author in the final version. The name of any person who is deemed to have made a minor contribution can, however, appear in the Acknowledgments section of the article.

Please complete the table below to indicate the contributions of all named authors to the manuscript.

Author Full Name:	Specification of Contribution to the Manuscript:
Faxi Wang & Fei Sun	designed the study, analyzed the data, and wrote the manuscript
Jiahui Luo & Tiantian Yue	performed WB & PCR
Longmin Chen & Haifeng Zhou	performed FACS
Jing Zhang & Chunliang Yang	did animal experiments
Xi Luo & Qing Zhou	did seahorse and finished supplementary results
He Zhu & Jinxiu Li	did IP and CHIP
Ping Yang & Fei Xiong	analyzed the data
Qilin Yu & Huilan Zhang	designed the experiments
Wanguang & ZhangAimin Xu	created the graphical illustrations
Zhiguang Zhou & Qianjin Lu	interpreted data
Decio L. Eizirik	discussed experimental design and contributed to manuscript writing
Shu Zhang	conceptalized the entire study
Cong-Yi Wang	supervised the entire study

Please complete the table below to indicate the contributions of all named authors to the figures.

Figure 1:

Faxi Wang (Fig. 1A-C) , Jing Zhang (Fig. 1D&E) , Longmin Chen (Fig. 1F-I)

Figure 2:

Haifeng Zhou & Chunliang Yang (Fig. 2A-D)

Figure 3:

Fei Sun (Fig. 3A&B), Faxi Wang & Tiantian Yue (Fig. 3C-F)

Figure 4:

Zhang Jing, (Fig. 4A), Jiahui Luo (Fig. 4B-D), Fei Sun (Fig. 4E-G), Faxi Wang (Fig. 4H)

Figure 5:

Jiahui Luo (Fig. 5A-E), Faxi Wang (Fig. 5F)

Figure 6:

Xi Luo & Qing Zhou (Fig. 6A-F), He Zhu & Jinxiu Li (Fig. 6G)

Signed for and on behalf of the Author(s):	Print Name:	Date:
[signature]	Cong-Yi Wang	11/06/2019
